# *Notes from the Field*: Mpox Cluster Caused by Tecovirimat-Resistant Monkeypox Virus — Five States, October 2023–February 2024

**DOI:** 10.15585/mmwr.mm7340a3

**Published:** 2024-10-10

**Authors:** Crystal M. Gigante, Jade Takakuwa, Daisy McGrath, Chantal Kling, Todd G. Smith, Mengfei Peng, Kimberly Wilkins, Jacob M. Garrigues, Taylor Holly, Hannah Barbian, Alyse Kittner, Danielle Haydel, Emma Ortega, Gillian Richardson, Julie Hand, Jill K. Hacker, Alex Espinosa, Monica Haw, Chantha Kath, Meilan Bielby, Kirstin Short, Kimberly Johnson, Nelson De La Cruz, Whitni Davidson, Christine Hughes, Nicole M. Green, Nicolle Baird, Agam K. Rao, Christina L. Hutson

**Affiliations:** ^1^Division of High-Consequence Pathogens and Pathology, National Center for Emerging and Zoonotic Infectious Diseases, CDC; ^2^Public Health Laboratories, Los Angeles County Department of Public Health, Downey, California; ^3^Chicago Department of Public Health, Chicago, Illinois; ^4^Regional Innovative Public Health Laboratory, Rush University Medical Center, Chicago, Illinois; ^5^Louisiana Department of Health; ^6^Viral and Rickettsial Disease Laboratory, California Department of Public Health; ^7^Houston Health Department, Houston, Texas; ^8^New York City Department of Health & Mental Hygiene, New York, New York.

**Summary**

**What is already known about this topic?**

Tecovirimat is the first-line drug for treatment of orthopoxvirus infection (e.g., smallpox and mpox). Viral mutations that render the drug ineffective can develop during treatment.

**What is added by this report?**

A new cluster of mpox cases caused by tecovirimat-resistant monkeypox virus (MPXV) was detected among persons with no documentation of previous tecovirimat treatment over multiple months in five U.S. states.

**What are the implications for public health practice?**

Routine sequence surveillance is needed to detect and monitor resistance. To prevent development and spread of resistant MPXV, tecovirimat use outside of clinical trials needs to be consistent with CDC’s Investigational New Drug protocol for tecovirimat use.

The antiviral drug tecovirimat* has been used extensively to treat U.S. mpox cases since the start of a global outbreak in 2022. Mutations in the mpox viral protein target (F13 or VP37) that occur during treatment can result in resistance to tecovirimat^†^ (*1*,*2*). CDC and public health partners have conducted genetic surveillance of monkeypox virus (MPXV) for F13 mutations through sequencing and monitoring of public databases. MPXV F13 mutations associated with resistance have been reported since 2022, typically among severely immunocompromised mpox patients who required prolonged courses of tecovirimat (*3*–*5*). A majority of patients with infections caused by MPXV with resistant mutations had a history of tecovirimat treatment; however, spread of tecovirimat-resistant MPXV was reported in California during late 2022 to early 2023 among persons with no previous tecovirimat treatment (*3*). This report describes a second, unrelated cluster of tecovirimat-resistant MPXV among 18 persons with no previous history of tecovirimat treatment in multiple states.

## Investigation and Outcomes

A unique combination of F13 mutations (asparagine 267 deletion [N267del] and alanine-184-to-threonine substitution [A184T]) was identified in 20 specimens collected from 18 mpox patients in five states (California [five], Illinois^§^ [eight], Louisiana [two], New York [one], and Texas [two]) during October 6, 2023–February 15, 2024. During their incubation periods, two patients reported travel among states where the mutation had been identified, and two others reported travel to other states. Among 16 patients with available treatment history, none had documentation of receipt of tecovirimat before collection of the resistant sample. One patient with fewer than 10 large lesions (0.79 in [>2 cm] in diameter) was prescribed a standard (i.e., 14-day) course of tecovirimat after sample collection; the patient recovered. Among 17 patients for whom clinical data were available, signs and symptoms^¶^ at initial examination were consistent with other clade IIb infections: all 17 patients reported mild (or not severe) mpox disease, although two were hospitalized for pain management. In vitro testing of seven samples identified resistance to tecovirimat, with 177-fold to 583-fold increases in the half-maximal effective concentration (EC_50_)** when compared with a 2003 U.S. MPXV clade IIa reference strain. This activity was reviewed by CDC, deemed not research, and was conducted consistent with applicable federal law and CDC policy.^††^

Whole genome sequences from all 20 specimens were genetically distinct from those in the 2022–2023 tecovirimat-resistant California cluster (*3*), which belonged to sublineage B.1.17 and contained the N267del mutation but not A184T. Genomes from the 2023–2024 cluster formed a monophyletic cluster within sublineage B.1.20 (the dominant U.S. clade IIb lineage during late 2023–early 2024), indicating that the resistance mutations were acquired by a common ancestor predating the sequenced samples (Figure). Both N267del and A184T mutations were present at allele frequencies >88% across specimens from the same patient and among all patients, which is atypical for acquired resistance (*4**,**5*). Together, the presence of the resistant phenotype and the observation that 88%–100% of the MPXV population within affected patient samples carried the resistant allele indicate tecovirimat would likely have been ineffective among those patients.

**FIGURE:**
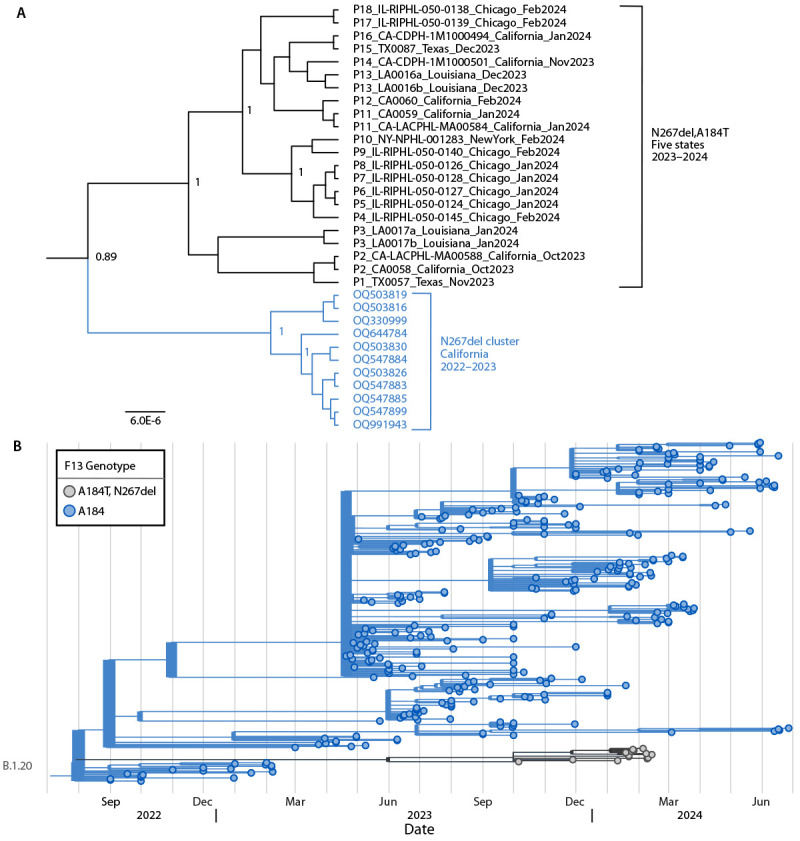
Phylogeny showing the relatedness of tecovirimat-resistant monkeypox viruses among 18 U.S. cases during 2023–2024*^,^^†^ with a previous tecovirimat-resistant cluster from California (A)^§^^,^^¶^ and with representative monkeypox virus sequences (B)** — United States, 2023–2024 **Abbreviations:** MPXV = monkeypox virus; A184T = alanine-184-to-threonine substitution; N267del = asparagine 267 deletion; SNP = single nucleotide polymorphism. * From five states (California, Illinois, Louisiana, New York, and Texas). ^†^ Unique patients are identified by P number; multiple samples were sequenced for four patients. ^§^ MPXV with N267del, A184T clustered separately from MPXV sequences from a tecovirimat-resistant cluster from California with N267del from late 2022–early 2023 (sublineage B.1.17). During late 2022–early 2023, persons in two of the four California cases (P16 and P2) reported travel to Texas; each had one or two SNP differences from an MPXV sequence from Texas (P15 and P1, respectively). Within the cluster, N267del, A184T sequences differed by an average of five SNPs (range = 0–11), compared with an average of 10.5 SNPs between the two clusters (range = 7–15). A large genomic deletion in sequence TX0087 was counted as one SNP. ^¶^ Phylogenetic analysis was performed using Bayesian Evolutionary Analysis Sampling Trees (BEAST; version 1.10.4) on whole genome sequence alignments of sequences after clipping genomic ends to match the shortest sequence, removing repeat regions, and removing any sites containing ambiguous bases to a final length of 150,505 positions using GTR+ G+I nucleotide substitution model, exponential coalescent tree prior, and strict molecular clock set to one; ON676708 was used to root the tree. Scale bar indicates substitutions per site; numbers at the branches indicate posterior probability support values. ** MPXV with N267del, A184T clustered separately from other sublineage B.1.20 MPXV sequences. Visualization of 334 lineage B.1.20 clade IIb MPXV genomes sampled from 3,552 genomes available from the National Center for Biotechnology Information GenBank between September 2022 and June 2024 (updated July 22, 2024) using a local build of Nextstrain (https://nextstrain.org) and visualized using TimeTree (https://timetree.org).

## Preliminary Conclusions and Recommendations

This is the second report of a tecovirimat-resistant MPXV variant spreading among persons in the United States who had no documentation of previous tecovirimat treatment and the first report of interstate spread. Because not all viruses from mpox cases are sequenced, these findings likely underestimate the prevalence of this newly recognized drug-resistant variant. This study calls attention to a need for increased sequence surveillance to determine whether the resistant virus is still circulating. The findings also underscore the importance of adhering to the CDC Investigational New Drug protocol for tecovirimat use outside of a clinical trial (i.e., indications for tecovirimat use, taking the recommended number of pills according to the prescribed schedule, and following instructions to take the medication with a fatty meal^§§^) and the importance of preventing spread^¶¶^ of a potentially resistant virus to others. The findings of this study and the PALM007 study*** highlight the urgent need for additional therapeutics for treatment of mpox as well as for smallpox biothreat preparedness.

## Footnotes

*
https://www.siga.com

† Orthopoxvirus VP37 amino acid substitutions associated with tecovirimat resistance compiled by the Food and Drug Administration from independent animal and cell culture studies and in a case of progressive vaccinia. https://www.fda.gov/emergency-preparedness-and-response/mcm-issues/fda-mpox-response

§ All Illinois cases were reported from Chicago.

¶
https://www.cdc.gov/mpox/signs-symptoms/index.html

** MPXV cultured from patient specimens were used to infect confluent Vero E6 cell monolayers pretreated with different concentrations of tecovirimat. After incubation for 72 hours, cells were fixed and stained with formalinized crystal violet and absorbance was measured at 570 nm; intact cell monolayers have a high absorbance indicating that the drug was protective; resistant MPXV requires higher concentrations of tecovirimat to inhibit virus and allow cell growth. EC_50_ was determined using a nonlinear curve fit in GraphPad Prism.

§§
https://www.cdc.gov/mpox/hcp/clinical-care/tecovirimat.html

¶¶
https://www.cdc.gov/mpox/prevention/index.html

*** The antiviral tecovirimat is safe but did not improve clade I mpox resolution in Democratic Republic of the Congo (https://www.nih.gov/news-events/news-releases/antiviral-tecovirimat-safe-did-not-improve-clade-i-mpox-resolution-democratic-republic-congo), although U.S. data from the NIH-sponsored Study of Tecovirimat for Human Mpox Virus trial will be essential in informing the role of tecovirmat for mpox treatment. https://www.clinicaltrials.gov/study/NCT05534984
